# Tombstoning ST-Elevation Myocardial Infarction

**DOI:** 10.2174/157340309789317869

**Published:** 2009-11

**Authors:** Bahattin Balci

**Affiliations:** Department of Cardiology, Van Specialist Trainig Education & Research Hospital, Van, Turkey

**Keywords:** Tombstoning, myocardial infarction, ST segment elevation

## Abstract

Tombstoning ST elevation myocardial infarction can be described as a STEMI characterized by tombstoning ST-segment elevation. This myocardial infarction is associated with extensive myocardial damage, reduced left ventricle function, serious hospital complications and poor prognosis. Tombstoning ECG pattern is a notion beyond morphological difference and is associated with more serious clinical results.

Despite the presence of a few reports on tombstoning ST elevation, there is no report which reviews STEMI demonstrating this electrocardiographic pattern.

## INTRODUCTION

Electrocardiography (ECG) is a complementary method in diagnosis and provides the risk stratification of people suspected of having myocardial infarction. It is vital in the classification of myocardial infarction [1]. Currently, myocardial infarction is classified in 2 groups: ST-elevation myocardial infarction (STEMI) and non-STEMI. STEMI is not a homogenous group in itself. Instead, differences are observed between patients in terms of various parameters such as the amplitude and morphology of ST-segment elevation, T-wave variations, the presence or absence of Q-wave, and clinical course and prognosis. Tombstoning ST-segment elevation is a type of ST-segment elevation with a specific morphology which is observed in the early period of acute myocardial infarction [2-7]. This ECG appearance is a notion beyond morphological difference and is associated with more serious clinical results. While there are a few reports on tombstoning ST elevation, there is no report which reviews STEMI displaying such ECG pattern. The aim of this report was to outline the profile of Tombstoning ST-elevation myocardial infarction (TOMB-STEMI) by reviewing the ECG, clinical, laboratory, and angiographic characteristics in detail.

## ECG CHARACTERISTICS

An ST-segment elevation with a specific pattern is the principal element of TOMB-STEMI. ST-segment elevation is often the earliest detected sign of acute MI. Initially, the ST segment may straighten, with loss of the ST-T wave angle. Then the T wave becomes broader and the ST segment elevates, losing its normal concavity. As further elevation occurs, the ST segment tends to become convex upwards [8]. As ST-segment elevation can be minimal, in some cases, it may surpass the peak level of the R wave. Thus, ST-segment elevation surpassing the R wave exhibits such a morphological appearance that it reminds a tombstone. Two electrophysiological mechanisms play a role in the formation of a tombstone appearance: delayed transmural conduction and intramyocardial conduction block [9]. The presence of tombstoning ECG in pericarditis and hypothermia suggests that it is not an infarction-specific event and that electrophysiological mechanisms may be involved in the formation of similar morphologies [10,11].

Wilmalaratna was the first to term this characteristic as tombstone-like and convex ST segment elevation in MI patients as tombstoning [2]. Later on, Guo *et al*. modified the tombstoning criteria [3]. The criteria of tombstoning ST-segment elevation are as follows: a) Absent R wave or an R wave duration <0.04 s with minimal amplitude, b) convex upward ST segment merging with the descending R or the ascending QS/QR, c) the peak of the ST segment is higher than the R wave and d) the ST segment merges with the T wave.

**Fig. (1):**
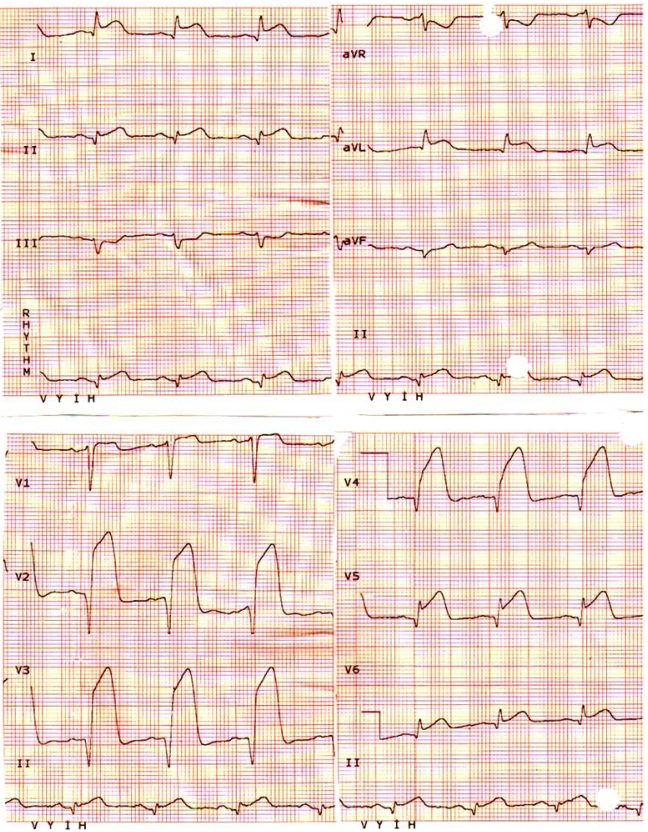
ST segment elevation meeting the criteria for tombstoning ECG.

**Fig. (2):**
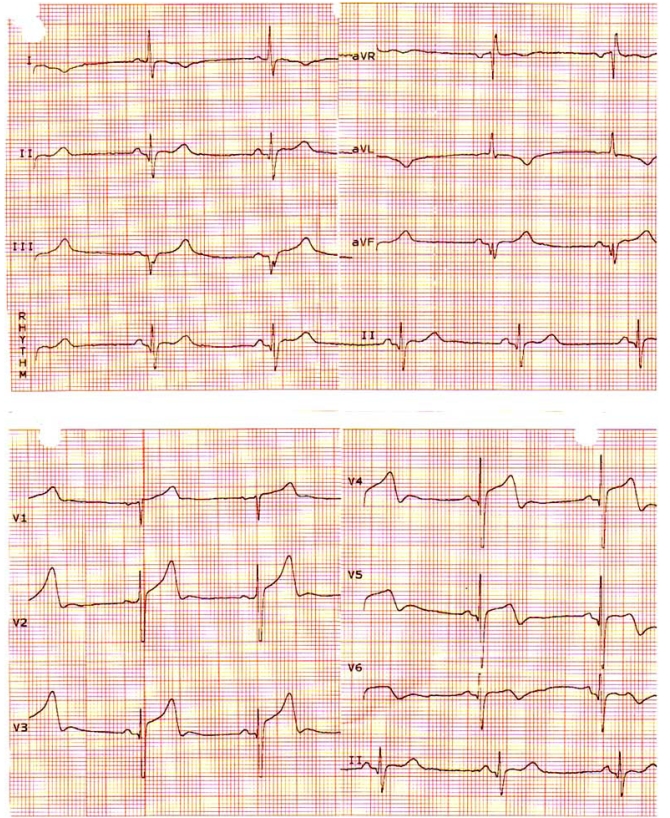
ST segment elevation not meeting the criteria for tombstoning ECG.

It is very easy to recognize and discriminate tombstoning ECG from others (Figs. **1** and **2**). The magnitude of ST elevation is determined fundamentally by the severity of epicardial damage. Nevertheless, amplitude of the ST elevation is affected by myocardial zone, chest structure, and distance of the electrode to the myocardial zone [14-16]. Tombstoning ECG in STEMI is not a rare event and observed among 10-26.1% of the patients [2-5]. While this kind of infarction is more commonly seen in anterior localization (39.8%), it may also be observed in inferior localization (10.6%) [6].

## ANGIOGRAPHIC CHARACTERISTICS

Regarding the coronary angiography findings of patients with TOMB-STEMI, the results of the studies are not consistent (Table **1**). Tomcyanyi *et al*. reported coronary disease incidence (2 or 3 vessel disease; 48% vs 54%; p=0.6311) and LAD involvement (LAD segment; 1.35 vs 1.5; p=0.44) in patients with tombstoning STEMI as similar to those of patients with STEMI who do not demonstrate this pattern [6]. However, Guo *et al*. suggested that patients with tombstoning have severe occlusion of the LAD artery (Total/partial occlusion of LAD; 100% vs 44%; p<0.0001) and usually involving either left circumflex or right coronary artery (3 vessel disease; 54.1% vs 22%; p=0.001) but more often both [3]. These different results in epicardial coronary anatomy might stem from the specific methodologies used by the studies. The fact that tombstoning is more commonly found in anterior than non-anterior STEMI may explain the higher rates of LAD disease in patients with tombstoning. Moreover, higher TIMI frame count and lower TIMI myocardial perfusion grade in the first one indicates the presence of a more severe ischemia in TOMB-STEMI cases [6].

## CLINICAL CHARACTERISTICS

Clinical and laboratory characteristics of patients with TOMB-STEMI are shown in Table **2**, whereas clinical profiles are shown in Table **3**. Coronary risk factors are similar in patients with and without the tombstoning pattern. Chest pain durations are similar. However, the incidence of preinfarct angina (39 vs. 64, p<0.03) is significantly lower [4]. Creatinine kinase (i.e.: 397 vs. 290, p<0.02) and brain natriuretic peptid (5 times higher) levels are elevated, and left ventricle ejection fraction (i.e.: 42 vs. 51, p<0.03) is reduced [4-6]. In other words, infarction size is larger and left ventricle function is worse in these patients.

## PROGNOSIS

Wilmanaratma associated tombstoning ECG pattern with the following complications. Six patients with tombstoning ECG pattern experienced ≥3 complications and 4 patients died within 7 days.^2^ Huang *et al*. reported reduced LV function and high mortality in patients with tombstoning ECG pattern [7]. Mortality in TOMB-STEMI is 26-38.2% [4,5]. As atrial arrhythmias are similar, life-threatening ventricular arrhythmias occur in a larger percentage of patients with TOMB-STEMI. This suggests the presence of variations in the ventricular arrhythmogenic substrate among TOMB-STEMI. Higher mortality rates may be explained with reduced pump function, life-threatening ventricular arrhythmias, and less reperfusion benefit.

Since it was defined, tombstoning ST-segment elevation has been associated with poor prognosis, but the reasons are still not fully understood. A few complementary hypotheses have been proposed. Several of the proposed mechanisms are as follows: extremely rapid myocardial damage, poor collateral flow and/or diffuse coronary artery disease, inadequate myocardial protection effect of preinfarct angina, and elevation of wall tension [3-6]. These hypotheses are based on severe ischemia, unprepared myocardium and resultant extensive myocardial damage. Preinfarct angina is associated with coronary collateral development and ischemic preconditioning [12]. Poor collateral flow or ischemic preconditioning in TOMB-STEMI supports the notion of unprepared myocardium. In other words, an unprepared, large myocardial area is exposed to severe ischemia, which leads to infarction displaying this ECG pattern.

## ECG IN RISK STRATIFICATION

Many studies have investigated application of 12-lead ECG for risk stratification. Various ECG variables have been studied such as terminal QRS (e.g.: Sclarovsky-Bimbaum score), ST segment (e.g.: ∑ST), T wave (e.g.: T wave inversion), and initial QRS developing during ischemia (e.g.: Selvester QRS score) [13-17]. Distortion of the terminal portion of QRS complex (grade III ischemia) is one of the ECG signs that are used to determine patients under high risk. Main criteria applied for terminal QRS distortion include disappearance of S wave in leads with RS morphology and a J point elevated above the lower half of R wave in leads with QR morphology. As patients with grade III ischemia demonstrate poor prognosis and larger final infarct size, they benefit less from thrombolytic treatment and primary angioplasty [13,14]. Grade III ischemia and TOMB-STEMI display similarities with respect to poor prognosis and less efficient reperfusion therapy.

Morphological changes occurring in the ECG are also included in the risk stratification analysis. Regardless of the total amplitude of the ST-segment elevation, tombstoning pattern has been proposed to be associated with higher mortality and ST elevation pattern has been reported to be a more important factor than quantitative changes (e.g: ∑ST) in risk stratification [5]. Pattern of the ST elevation has been shown to be a strong prediction factor in acute MI. While concave ST elevation is associated with perfect LV function, convex ST elevation is associated with poor LV function [18]. Along with the quantitative changes in ST segment elevation, inclusion of morphologic alteration in risk stratification may contribute in obtaining more consistent results. 

## CONCLUSIONS

It appears that a sudden occlusion of a coronary artery supplying a large area of unprepared myocardium; i.e. myocardium not protected by collaterals or ischemic preconditioning, results in complete transmural injury rapidly progressing to complete infarction, resulting in this characteristic ECG pattern. The extensive nature of the myocardial infarction and the resultant left ventricular damage and dysfunction may explain the higher risk of complications and mortality associated with this finding. The higher BNP levels on presentation in patients with tombstone STEMI seem to support the extensive nature of the myocardial damage associated with this ECG finding.

What needs to be further defined? It is not well defined yet whether most of these patients progress to rapid and irreversible Q wave formation on the ECG, compared to patients without this ST elevation pattern. If the damage is rapid and extensive, it is unclear whether early percutaneous revascularization results in adequate myocardial salvage of patients with tombstoning, when compared to patients without this finding. Follow-up studies of these patients after revascularization should help answer such a question. Tissue edema, microvascular plugging and inflammation that follow such extensive and rapid necrosis may explain the slower TIMI frame count, poor TIMI myocardial perfusion grade and the higher incidence of unsuccessful PCI. If myocardial recovery, despite revascularization, is shown to be limited in these patients, then additional intracoronary pharmacological measures; i.e. intracoronary adenosine, GP IIb/IIIa inhibitors and/or postconditioning measures (serial 1 min balloon occlusion of the coronary artery during PCI followed by reperfusion by balloon deflation) may be necessary to improve outcome in these patients. Since approximately 25% of patients with anterior STEMI have this finding on the ECG, a better definition of the significance of tombstoning STEMI is urgently needed and may result in more aggressive measures that aim myocardial salvage in these patients.

Further studies are required to reveal the mechanism of tombstoning ST-segment elevation, its relations with complications and its effects on long-term prognosis as well as optimization of medical or invasive treatments for TOMB-STEMI. Because this myocardial infarction is more than a mere ECG pattern with a specific morphological appearance, evaluating it as a different entity seems to be appropriate.

## Figures and Tables

**Table 1:** Angiographic Characteristics of TOMB-STEMI

Parameters	Guo *et al.* [3]	Tomcsanyi *et al*. [6]
Total/partial occlusion of LAD, %	100 vs 44, p<0.0001	
LAD =100% occlusion,%	50 vs 20.5, p=0.02	
LAD< 50% occlusion ,%	0 vs 15.9, p=0.039	
Proximal occlusion of LAD, %	92 vs 65, p=0.017	
3 vessel disease,%	54.1 vs 22, p=0.001	
2 or 3 vessel disease,%		48 vs 54, p=0.6311
LAD segment		1.35 vs 1.5, p=0.44
Open culprit artery,%		26 vs 34, p=0.284
TIMI frame count		28 vs 17.2, p=0.0001
TIMI myocardial perfusion grade		1.2 vs 1.8, p=0.043
Unsuccessful PCI, %		22 vs 6, p=0.05

LAD=left anterior descending coronary artery; TIMI=thrombolysis in myocardial infarction; PCI=percutaneous coronary intervention.

**Table 2:** Clinical and Laboratory Characteristics of TOMB-STEMI

Parameters	Balci *et al*. [4]	Kukla *et al*. [5]	Tomcsanyi *et al*. [6]
TC,mg/dl	197 vs 194, p=0.70		
LDL,mg/dl	135 vs 126, p=0.30		
HDL,mg/dl	34 vs 37, p=0.30		
DM, %	25 vs 18, p=0.37	22.4 vs 14.6, NS	26 vs 18, p=0.55
HT, %	34 vs 26, p=0.26		53 vs 48, p=0.81
Smoker, %	62 vs 61, p=0.91		
Chest Pain, hours		4,7 vs 4.9, NS	
Preinfarct angina , %	39 vs 64, p<0.03		
SBP, mmHg	101 vs 116,p0.05		
DBP, mmHg	64 vs 73, p=0.06		
CK-MB, IU/L	397 vs 290, p<0.02		
Peak CK, IU/L		1598.9 vs 1575, NS	
EF, %	42 vs 51, p<0.03	40.9 vs 48.6, p=0.001	42 vs 45, p=0.2
Death, %	26 vs 2, p<0.01	38.2 vs 9.9, p=0.001	13 vs 6, p=0.37
Cardiogenic shock, %	22 vs 2, p<0.02	21.8 vs 12.3, NS	9 vs 3, p=0.26
Heart failure, %		45.6 vs 28.3, p=0.026	22 vs 14, p=0.50
Pulmonary oedema, %		14.5 vs 9.6, p= NS	
Ventricular tachycardia, %	17 vs 2, p<0.01		
sVT/VF, %			9 vs 6, p=0.65
Ventricular fibrillation, %	30 vs 5, p<0.02	18.1 vs 6.4, p=0.016	
Atrial fibrillation, %	4 vs 2, p=0.52		
AV block, %	13 vs 10, p=0.44		

TC=total cholesterol; LDL= low-density lipoprotein; HDL= high-density lipoprotein; DM=diabetes mellitus; HT=systemic hypertension; SBP =systolic blood pressure; DBP=diastolic blood pressure, CK=creatine kinase; EF=left ventricular ejection fraction; sVT/VF=sustained entricular tachycardia/ ventricular fibrillation; AV=atriovenricular NS=nonsignificant.

**Table 3:** Clinical Profile of TOMB-STEMI Compared with Non-TOMB-STEMI

Coronary risk factors	Similar
Symptom	Similar angina pectoris but less frequent preinfarction angina
Laboratory	Higher CK, higher BNP which means larger infarction area.
Echocardiography	Lower left ventricle EF which means a heavier left ventricle dysfunction
Angiography	Similar epicardial coronary anatomy or more extensive. Higher TIMI frame count and lower TIMI myocardial perfusion grade which means more severe ischemia
Complication	More complications and poor prognosis
Reperfusion	Less efficient reperfusion therapy

CK=creatine kinase; BNP=brain natriuretic peptid; EF= ejection fraction; TIMI=thrombolysis in myocardial infarction.
